# IS*Apl4*, a New IS*1595* Family Insertion Sequence Forming a Novel Pseudo-Compound Transposon That Confers Antimicrobial Multidrug Resistance in *Actinobacillus pleuropneumoniae*

**DOI:** 10.3390/antibiotics14101021

**Published:** 2025-10-14

**Authors:** Janine T. Bossé, Yanwen Li, Marc Stegger, Liza Miriam Cohen, Øystein Angen, Søren Overballe-Petersen, Dennis Hanke, Stefan Schwarz, Paul R. Langford, Henrike Krüger-Haker

**Affiliations:** 1 Section of Paediatric Infectious Disease, Department of Infectious Disease, Imperial College London, South Kensington Campus, London SW7 2AZ, UK; j.bosse@imperial.ac.uk (J.T.B.); yanwen.li@imperial.ac.uk (Y.L.); p.langford@imperial.ac.uk (P.R.L.); 2 Department of Sequencing and Bioinformatics, Statens Serum Institut, 2300 Copenhagen, Denmark; mtg@ssi.dk; 3 Antimicrobial Resistance and Infectious Diseases Laboratory, Harry Butler Institute, Murdoch University, Murdoch, Perth, WA 6150, Australia; 4 Department of Production Animal Clinical Sciences, Faculty of Veterinary Medicine, Norwegian University of Life Sciences (NMBU), NO-1432 Ås, Norway; liza.miriam.cohen@nmbu.no; 5 Department of Bacteria, Parasites and Fungi, Statens Serum Institut, 2300 Copenhagen, Denmark; ysan@ssi.dk (Ø.A.); sovp@ssi.dk (S.O.-P.); 6 Institute of Microbiology and Epizootics, School of Veterinary Medicine, Freie Universität Berlin, 14163 Berlin, Germany; dennis.hanke@fu-berlin.de; 7 Veterinary Centre for Resistance Research (TZR), School of Veterinary Medicine, Freie Universität Berlin, 14163 Berlin, Germany

**Keywords:** *Actinobacillus pleuropneumoniae*, antimicrobial resistance, IS*1595*, IS*1016*, IS*Apl4*, pseudo-compound transposon, Tn*7560*, transformation

## Abstract

Background/Objectives: *Actinobacillus pleuropneumoniae* is an important respiratory tract pathogen of swine worldwide. Insertion sequences (ISs) play a major role in the transfer of antimicrobial resistance (AMR) among various porcine respiratory tract pathogens. In this study, three *A. pleuropneumoniae* genomes were investigated for the presence of a novel IS. Methods: Analysis of the draft genomes of three *A. pleuropneumoniae* serovar 8 isolates (AP_1, AP_120, AP_123) suggested the presence of a novel IS. A closed whole-genome sequence was generated for strain AP_123 by hybrid assembly of Oxford Nanopore MinION long-reads and Illumina MiSeq short-reads, followed by sequence analysis using standard online tools. Transfer was tested by natural transformation. Antimicrobial susceptibility testing was conducted by broth microdilution following Clinical and Laboratory Standards Institute standards. Results: A novel IS, designated IS*Apl4*, was detected in all three genomes. IS*Apl4* is 712 bp in size and has a transposase gene (*tnp*) of 654 bp. Moreover, it has perfect terminal 14-bp inverted repeats and produces 8-bp direct repeats at its integration site. This IS was found in 39 copies in the AP_123 genome, two of which formed the 5,765-bp pseudo-compound transposon Tn*7560*. This transposon carries four AMR genes: *sul2* (sulfonamide resistance), *strA-strB* (streptomycin resistance), and *tet*(Y) (tetracycline resistance). RT-PCR confirmed *tnp* gene expression and horizontal transfer of Tn*7560* into *A. pleuropneumoniae* MIDG2331. Conclusions: This study identified the novel IS*Apl4* in porcine *A. pleuropneumoniae* and its association with the novel pseudo-compound transposon Tn*7560*, which proved to be an active element capable of disseminating multidrug resistance amongst *A. pleuropneumoniae*.

## 1. Introduction

Horizontal transfer of antimicrobial resistance (AMR) genes amongst bacteria that infect livestock not only has an impact on the costs of food animal production but also increases the risk of transmission of resistant bacteria among and between animals, as well as to humans [1,2,3]. *Actinobacillus pleuropneumoniae*, a member of the *Pasteurellaceae* family, is one of the most important respiratory pathogens of swine and is responsible for major economic losses worldwide [4]. Despite increased regulatory restrictions in many countries, control of porcine pleuropneumonia still depends on the judicious use of antimicrobial agents during outbreaks, as commercially available vaccines are not fully efficacious against all *A. pleuropneumoniae* serovars and do not prevent colonization [5]. Levels of resistance to various antimicrobial agents differ between countries, reflecting preferred treatment regimens (e.g., choice of antimicrobial agents, dosage, and treatment duration). For example, several countries have reported high levels of tetracycline resistance in *A. pleuropneumoniae* [6,7], whereas in Denmark and Norway, most isolates remain susceptible to this agent [8,9].

There are more than 50 known genes that confer resistance to tetracyclines (http://faculty.washington.edu/marilynr/ (accessed on 29 September 2025)). However, until recently, only eight were found in *A. pleuropneumoniae* and other *Pasteurellaceae* species of veterinary origin, including *tet*(A), *tet*(B), *tet*(C), *tet*(G), *tet*(H), and *tet*(L), which encode efflux proteins, as well as *tet*(M) and *tet*(O), which encode ribosome protective proteins [1]. Of these, most have been found on plasmids and/or integrative and conjugative elements (ICEs), and some have been further associated with other mobile genetic elements (MGEs), such as transposons and insertion sequences (ISs) [1]. Most recently, a ninth tetracycline resistance gene, *tet*(Y), which so far has mainly been seen in various Gammaproteobacteria, including *Escherichia coli*, *Salmonella enterica*, *Acinetobacter* spp., and *Moraxella* spp., has been identified on several novel multidrug-resistance (MDR)-mediating ICEs in porcine and bovine *Pasteurella multocida* [10,11] as well as bovine *Mannheimia haemolytica* [12] from Germany, but not yet in *A. pleuropneumoniae*.

ISs are small MGEs, typically ≤3 kb, that generally only carry a gene for a transposase required for their movement between genetic locations. They are a ubiquitous and diverse type of MGEs, which are found in most bacterial genomes and can be grouped into different families based on common features, such as (i) the type of transposase (with catalytic site conservation), (ii) the similarity of the terminal inverted repeats, (iii) the target-site preference, and (iv) the length of the flanking repeats (i.e., the target site duplications) generated upon insertion [13,14]. Currently, there are at least 26 families of ISs, some of which can be further differentiated into subgroups with an ever-expanding database, ISfinder (https://www-is.biotoul.fr (accessed on 29 September 2025)), as new elements are discovered in the growing number of publicly available bacterial genomes. The transposition of ISs can either have a neutral effect on the genome or result in insertions that either increase or decrease bacterial fitness [15,16]. Regulation of transposition by IS- and/or host-mediated mechanisms is common, and IS copies may be eliminated from the genome via deletions and rearrangements [13]. Loss of ISs may leave “scars”, i.e., partial sequences lacking intact *tnp* genes and/or flanking inverted repeat (IR) sequences, and many bacterial genomes show evidence of ancestral transposition events [13].

Selective pressure for the dissemination of IS elements can occur through the formation of compound (also known as composite) transposons or pseudo-compound transposons, both carrying beneficial genes, such as those conferring AMR, flanked by two copies of the IS element [17,18]. Based on the orientation of the terminal ISs, compound (or composite) transposons and pseudo-compound transposons are differentiated [18]. While the ISs in the first type of transposons are in opposite orientation, structures bounded by directly oriented ISs are referred to as pseudo-compound transposons [18]. Despite the initial definition only including members of the IS*26* family [18], it may also apply to members of other IS families. After compound transposons are acquired by horizontal transfer into a host bacterium, they can transpose into different locations in the chromosomal DNA or plasmids (if present) as an intact transposon. Alternatively, each copy of the IS element can further transpose separately [15]. This can lead to a rapid expansion of IS copies in a genome, with possible subsequent elimination as discussed above. In the case of pseudo-compound transposons, the two terminal ISs can recombine and form a translocatable unit (TU) that consists of the part between the two ISs and one copy of the IS [19]. This short-lived circular intermediate can again recombine with other copies of the respective IS either in the chromosomal DNA or on plasmids and, thereby, promote its change of location within the same cell. If, however, a TU integrates into an ICE, it can be exchanged across strain, species or genus boundaries as part of this conjugative MGE [20].

Three IS elements have been reported previously in *A. pleuropneumoniae.* IS*Apl1*, a functional IS*30* element, was identified in serovar 7 isolates [21]; whereas IS*Apl2* and IS*Apl3* (which are 98% identical) are members of the IS*3* family but are predicted to be non-functional due to an internal frameshift mutation in the *tnp* gene. The former was from an unidentified isolate, the sequence of which was deposited directly in the ISfinder database, and the latter was identified in the genome of the serovar 5 reference strain L20 (https://www-is.biotoul.fr/list_names_attributed.php (accessed on 29 September 2025)). In a comparative analysis of draft genome sequences of *A. pleuropneumoniae* serovar 8 isolates from the United Kingdom (UK), Denmark, and Norway [8], three Norwegian isolates (AP_1, AP_120, and AP_123) were identified as the first *A. pleuropneumoniae* isolates carrying the *tet*(Y) gene along with other resistance genes. Further analysis of their draft genomes suggested the presence of a novel IS, designated IS*Apl4*. In this study, the structure and function of IS*Apl4*, as well as a novel pseudo-compound MDR transposon, carrying IS*Apl4* copies in the same orientation at its termini, were investigated in detail.

## 2. Results

### 2.1. Identification of ISApl4

In the draft genomes of the three Norwegian *A. pleuropneumoniae* isolates of serovar 8 (AP_1, AP_120, and AP_123), we identified an IS of 712 bp that carried a single reading frame of 654 bp encoding a predicted transposase of 217 aa. This IS, designated IS*Apl4*, was bounded by perfect IRs of 14 bp (5′-GGGGCTGTACTAGA-3′). A sequence with the greatest identity of 99.58% (=709/712 bp) to IS*Apl4* was detected by blastn in the genomes of two *Moraxella* spp. isolates, FZLJ2107 and FZLJ2109, from pigs in China (GenBank accession nos. CP101111 and CP101112), each of which carried three identical copies of this IS*Apl4*-related element. There were three differences at the nucleotide sequence level seen in comparison to IS*Apl4*, all of which were located within the *tnp* reading frame. Two of them, G233A and G262A, changed the aa sequence to R78H and G88S, respectively, while the third, C276T, did not affect the V at position 92 in the deduced transposase protein sequence. Slightly lower nucleotide sequence identities, all ranging between 95.51% and 91.29% were seen with various *Moraxella* isolates, including *Moraxella veridica* ATCC 23246 (GenBank accession no. CP170124), *Moraxella canis* of not further specified origin from Finland (GenBank accession no. CP139961), several bovine *Moraxella bovoculi* isolates from China (GenBank accession nos. CP195518 and CP195440) or the United States of America (USA) (GenBank accession nos. CP011381, CP011380, CP011379, CP011378, CP011377, CP011376, and CP011374), and a *Moraxella ovis* isolate that was recovered from a cow in Norway already in 1959 (GenBank accession no. CP011158).

In contrast, the blastp analysis indicated that the IS*Apl4*-encoded transposase protein shares 100% identity with IS*1016* subgroup transposases of *Mannheimia haemolytica* from a cow in the Netherlands (GenBank accession no. HDL1205338), or isolates of *P. multocida* from cattle in Australia (GenBank accession nos. MDY0626729, MDY0678315, MDY0682612, and MDY0710592), from turkeys in the USA (GenBank accession nos. HDR1109752 and HEA3246051), and from a pig in the USA (GenBank accession no. HEH9724036). The next-related IS*Apl4*-encoded transposase proteins are those from *Moraxella* spp. isolates FZLJ2107 and FZLJ2109 with 99.08% (215/217 aa). The discrepancy between the blastn and blastp results might be because for some protein sequences, the associated nucleotide sequences are not available in the databases to which blastn has access.

Further inspection of the IS*Apl4* sequence revealed that it is a DDE transposase, having a predicted catalytic site sequence, E-**D**ESYFG_(53)_VYT**D**----Y_(24)_HINGI**E**NFWSQA**K**_(14)_LFIK**E**CEFRFN, characteristic of transposases of IS*1016* elements, which are a subfamily of IS*1595* elements [22,23].

Sequences matching either the 5′ or 3′ ends of the 712-bp sequence were mapped to multiple contigs in each of the AP_1, AP_120, and AP_123 draft genomes. By comparison to the complete closed genomes of other serovar 8 strains 405 and MIDG2331, mapping of the partial 5′ and 3′ ends of the IS*Apl4* element (with regard to flanking genes) indicated 18 IS*Apl4* insertions in AP_1, with 16 of these also seen in AP_120, along with a further nine insertions not found in AP_1. The AP_123 genome contains 39 IS*Apl4* insertions, with only four (including the two that represent part of a pseudo-compound transposon) being the same as in the other two isolates, as well as three in the same location, but opposite orientation.

**Figure 1:**
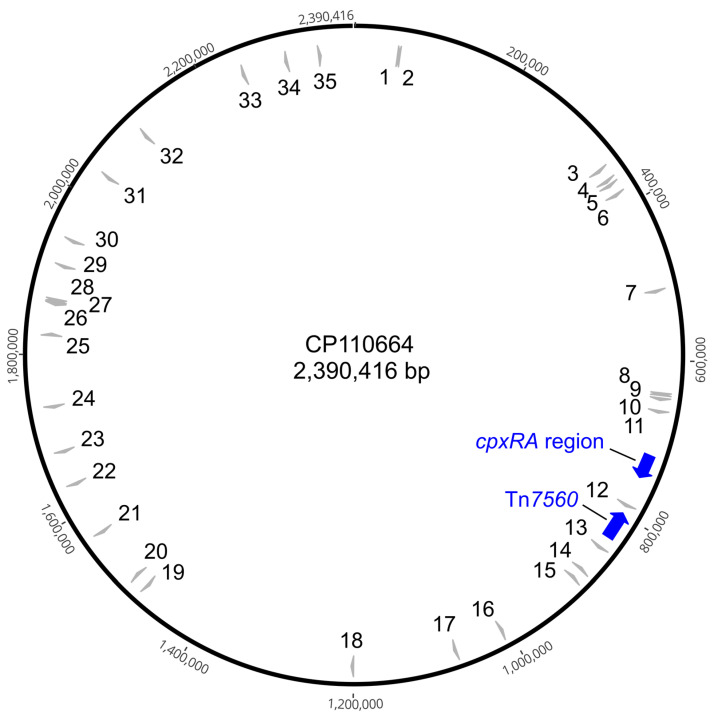
Mapping of 35 of the 39 IS*Apl4* copies across the closed genome of isolate AP_123 (GenBank accession no. CP110664). A size scale is given outside the circular map. The positions of the novel transposon Tn*7560* and the *cpxRA* region, respectively, are indicated by blue arrows.

To confirm the insertion sites predicted for the draft genomes, and to determine if any recombination events might have been mediated by the multiple copies of IS*Apl4*, a closed genome sequence was generated for AP_123 (GenBank accession no. CP110664), the isolate containing the highest number of insertion sites (Figure 1). The predicted insertion sites detected in the draft genome were confirmed in the closed genome (Table 1). There was only one rearrangement detected, i.e., an inversion of a sequence containing the *cpxR* gene and most of the *cpxA* gene found between two closely spaced copies of IS*Apl4*. The CpxRA system controls the expression of genes involved in biofilm formation [24,25,26,27].

Only the closed AP_123 genome allowed confident mapping of both flanks of each insertion. Detailed analysis of the integration sites in AP_123 revealed that IS*Apl4* produced preferably A/T-rich 8-bp direct repeats (DRs) immediately up- and downstream of its integration sites (Table 1). This observation is in agreement with the 7–9-bp DRs seen with other IS*1016* elements [22]. These 8-bp DRs were identified for all but four IS*Apl4* insertions, i.e., those associated with the pseudo-compound transposon and those associated with the inverted sequence in the *cpxRA* region.

Most mapped insertions, including those of IS*Apl4* copies 1–2, 4–7, 9–10, 14–15, 17, 21, 26–30, and 34–35 (Table 1), were located in non-coding intergenic regions, although some were within genes, including several known or predicted to contribute to virulence. Thus, IS*Apl4* copy 3 was located within a gene encoding a drug/metabolite transporter (DMT) family EamA domain-containing protein. IS*Apl4* copy 8 was found within the gene *tadA* encoding a CpaF family protein/tight adherence protein A. IS*Apl4* copy 11 was inserted into a gene encoding a sulfatase N-terminal domain-containing protein, and IS*Apl4* copy 12 was located in a gene encoding a tetratricopeptide repeat (TPR) protein. The gene *udk* encoding a uridine kinase was interrupted by the insertion of IS*Apl4* copy 16. IS*Apl4* copy 19 was inserted into the phosphate regulon transcriptional regulator gene *phoB*, while copy 20 was within a gene encoding a Tex family protein. IS*Apl4* copy 22 was inserted into the *manX* gene encoding the phosphotransferase system (PTS) mannose transporter subunit IIAB. IS*Apl4* copy 23 was detected within a gene encoding a lipopolysaccharide (LPS) O-antigen chain length determinant protein of the WzzB/FepE family. IS*Apl4* copy 24 inactivated the gene encoding an N-6 DNA methylase/class I S-adenosyl methionine (SAM)-dependent DNA methyltransferase, while IS*Apl4* copy 25 was found within the *wzxE* gene encoding the lipid III flippase WzxE, a LPS biosynthesis protein. IS*Apl4* copy 31 disrupted the *mutY* gene encoding an A/G-specific adenine glycosylase, and IS*Apl4* copy 32 was within a gene encoding a DUF1523 domain-containing protein. Finally, IS*Apl4* copies 13, 18 and 33 were inserted into genes for hypothetical proteins.

### 2.2. Analysis of the Tn7560 Sequence

**Figure 2:**
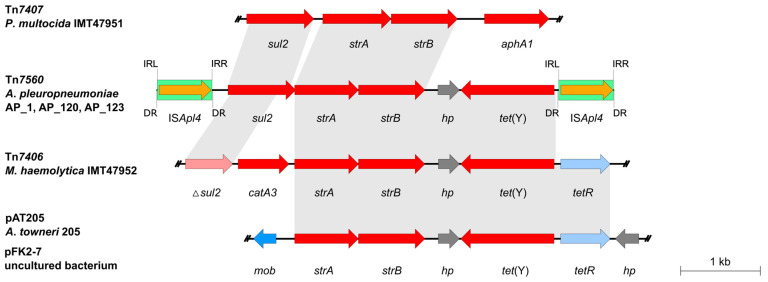
Structure and organization of transposon Tn*7560* in comparison with related elements. The open reading frames are shown as arrows with the arrowhead indicating the direction of transcription. Resistance genes are marked in red, transposase genes in orange, other genes in blue and hypothetical (*hp*) genes with unknown function in grey. The truncated *sul2* gene is indicated by a ∆ symbol. IS*Apl4* elements are displayed as green boxes. Identities of at least 99% between sequences are indicated by grey shading. A size scale is given on the right-hand side. IRL, inverted repeat left; IRR, inverted repeat right; DR, direct repeat.

Tn*7560* (https://transposon.lstmed.ac.uk/ (accessed on 29 September 2025)) is a pseudo-compound transposon of 5765 bp in size. The 4341-bp sequence between the two identical IS*Apl4* elements located in the same orientation harboured four different AMR genes: the sulfonamide resistance gene *sul2*, the streptomycin resistance genes *strA*-*strB*, and the tetracycline resistance gene *tet*(Y). The blastn analysis of this 4341-bp segment revealed the highest nucleotide sequence identities of ≥99.70% each to two segments, one comprising *tet*(Y), *strA* and *strB* and the other *sul2*, that are part of chromosomally located Tn*7406* and Tn*7406*-related ICEs of German bovine *P. multocida* (GenBank accession no. CP172107.1) [11] and *M. haemolytica* isolates (GenBank accession nos. CP128531.1 (Tn*7724*), CP128523.1 (Tn*7727*), and CP087379.1) [12,28]. Similarly high nucleotide sequence identities were detected in the 97,979-bp plasmid pB2126 and the chromosomal DNA of several *Pseudomonas* spp. isolates, all of fish product origin from France (GenBank accession nos. CP162591.1, CP162591.1, CP162581.1, CP162583.1) as well as in the 158,867-bp plasmid pAT205 of an *Acinetobacter towneri* isolate of swine faeces origin from China (GenBank accession no. CP048015) (Figure 2). The 3,265-bp sequence containing the *tet*(Y) and *strA*-*strB* genes is also 100% identical to sequences from other *Acinetobacter* species recovered in China (GenBank accession nos. CP029397 and CP010351), as well as part of the 48,493-bp low GC conjugative plasmid pFK2-7 (GenBank accession no. KT325596) from an uncultured bacterium isolated from cattle feces in the Czech Republic [29]. However, plasmid pFK2-7 does not encode either *sul2* or any IS*1595*-type *tnp* gene. Sequences identical to the *sul2*-*strA*-*strB* gene arrangement seen in Tn*7560* have been identified in other *Pasteurellaceae* ICEs, such as those seen in *P. multocida* isolates FCf83 and FCf71 (GenBank accession nos. CP038875 and CP038872) and *Glaesserella parasuis* isolates GHP1807 and YHP170504 (GenBank accession nos. CP071491 and CP054198).

In the Tn*7560* sequence, the *sul2* gene is 852 bp in size rather than the 816 bp present in most *sul2* GenBank entries. The reason for this is that the final “A” in the translational termination codon “TAA” is replaced by a “T”, thereby extending the *sul2* reading frame by 36 bp and generating an alternative stop codon. This expanded *sul2* reading frame overlaps the 5′ end of *strA* by 14 bp. The identical sequence has been found previously on plasmid pYFC1 from *M. haemolytica* [30]. Another interesting observation is the lack of the *tet*(Y)-associated *tetR* gene in Tn*7560*. Many *tet* genes specifying efflux-mediated tetracycline resistance in Gram-negative bacteria are regulated by the interplay between Tet repressor and Tet efflux proteins. This is also true for most known *tetR*-*tet*(Y) operons found in the aforementioned closely related sequences. Nevertheless, the *tet*(Y) gene in Tn*7560* seems to be functional as the respective *A. pleuropneumoniae* isolates AP_1, AP_120, and AP_123 exhibited tetracycline minimal inhibitory concentrations (MICs) of 8 or 16 µg/mL (Table 2), classifying them as tetracycline-resistant according to the Clinical and Laboratory Standards Institute (CLSI)-approved *A. pleuropneumoniae*-specific clinical breakpoints [31]. A previous report on the tetracycline resistance gene *tet*(L) also identified that this gene was functional in *M. haemolytica* and *Mannheimia glucosida* without its regulatory region [32].

The closed AP_123 genome also enabled mapping of the insertion site of Tn*7560* in a region upstream of the *dld* gene, which encodes a D-lactate dehydrogenase. As no insertion site could be mapped upstream of *dld* in AP_1 or AP_120, the transposon insertion site must be different in these isolates.

### 2.3. Single Nucleotide Polymorphism (SNP) Analysis

Comparing the genomes of AP_1, AP_120, AP_123, and 405, using the MIDG2331 chromosome as a reference, detected a total of 15,603 SNPs across all isolates in a 2.10 Mb core genome. MIDG2331 and 405 were separated by ~400 SNPs but differed by >15,000 SNPs compared to the three Norwegian isolates. Among the latter three isolates, AP_1 and AP_120 were almost identical, with only six SNP differences, whereas AP_123 contained between 111 and 113 SNP differences compared to AP_1 and AP_120, respectively. An in-depth analysis confirmed that the differences between the isolates of Norwegian origin were not due to recombination [8].

### 2.4. Confirmation of ISApl4 Activity

**Figure 3:**
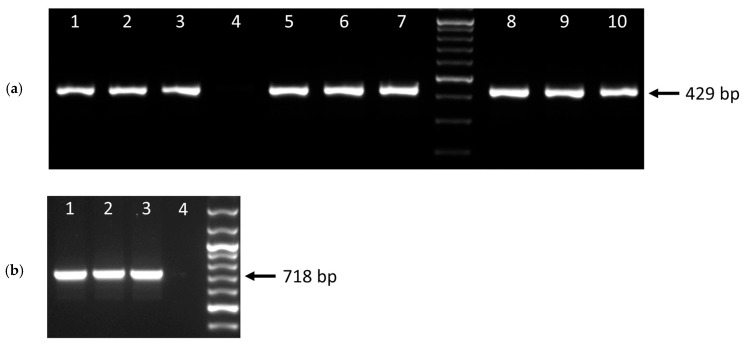
(**a**) Confirmation of expression of the IS*Apl4 tnp* gene in different *A. pleuropnemoniae* isolates, as determined by amplification of a 429-bp sequence. Lanes 1–3 show the results for AP_1, AP_120, and AP_123, respectively, grown in non-selective medium. Lane 4 shows no amplification from MIDG2331. Lanes 5–7 show the results for AP_1, AP_120, and AP_123, respectively, grown in medium containing 5 µg/mL tetracycline. Lanes 8–10 show the results for the MIDG2331 transformants obtained using genomic DNA (gDNA) from AP_1, AP_120, and AP_123, respectively, grown in medium containing 5 µg/mL tetracycline. (**b**) Confirmation of circular intermediate formation by IS*Apl4* during transposition. Inverse PCR was used to amplify a 718.bp product from each of AP_1, AP_120 and AP_123. Marker = GeneRuler 100 bp Plus DNA Ladder (Thermo Fisher Scientific, Loughborough, UK).

Expression of the *tnp* gene was confirmed by reverse transcription (RT)-PCR in AP_1, AP_120 and AP_123 (Figure 3a), indicating that the gene is actively transcribed in these isolates. The amplicons generated using RNA extracted from cultures grown in the presence of 5 µg/mL tetracycline appeared slightly more intense than those from cultures grown in non-selective medium; this difference was minor and not quantified. Inverse PCR confirmed the presence of a 718-bp circular intermediate of the IS*Apl4* element present in DNA extracted from each of AP_1, AP_120 and AP_123 (Figure 3b), indicating that the 8-bp DR sequences flanking the element facilitate excision during transposition.

Transfer of the pseudo-compound transposon to another strain of *A. pleuropneumoniae* was achieved via natural transformation under the conditions tested. Donor gDNA from each of AP_1, AP_120 and AP_123 resulted in MIDG2331 transformants, as confirmed by resistance to streptomycin. The MICs of tetracycline, sulfisoxasole, and streptomycin were the same for the transformants as for the original Norwegian isolates (Table 2). Expression of the *tnp* gene in selected transformants resulting from each donor strain’s gDNA was confirmed by RT-PCR, as for the original donor isolates above (Figure 3a).

**Figure 4:**
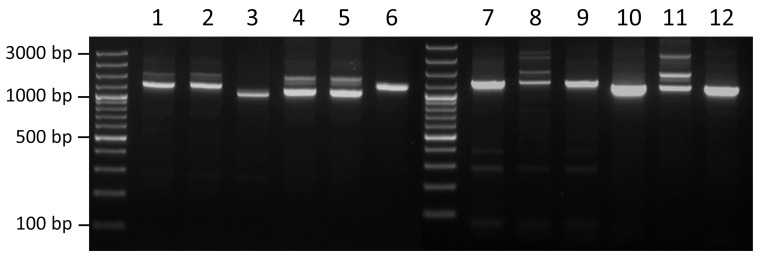
Results of linker PCR mapping of the insertion sites for Tn*7560* in different *A. pleuropneumoniae isolates*. Lanes 1–3 show the sul2_up_out amplicons, and lanes 4–6 show the tetY_5′_out for the original AP_1, AP_120, and AP_123 isolates, respectively. Lanes 7–9 show the sul2_up_out amplicons, and lanes 10–12 show the tetY_5′_out for the MIDG2331 transformants obtained using gDNA from AP_1, AP_120, and AP_123, respectively. Marker = GeneRuler 100 bp Plus DNA Ladder (Thermo Fisher Scientific).

Linker-PCR, using outward-facing transposon-specific primers, was used to map the location of the composite transposon in the AP_1 and AP_120 strains, as well as in the MIDG2331 transformants obtained using gDNA from AP_1, AP_120, and AP_123 (Figure 4). The multiple bands seen for AP_1 and AP_120, generated with both the tetY_5′_out and the sul2_up_out primers, suggest the presence of more than one copy of Tn*7560* in the genomes. Furthermore, the band patterns for these isolates appear to be the same, likely indicating the same insertion sites. It was not possible to obtain clean sequences showing the insertion sites for AP_1 or AP_120 due to the multiple amplicons generated by linker PCR. In contrast, AP_123 has only one copy of Tn*7560*, as indicated by the single amplicon for each flank in the linker PCR. Sequencing of these amplicons confirmed the insertion site, upstream of the *dld* gene, as seen in the closed genome for AP_123. The selected clone for the MIDG2331/AP_1 transformant showed that only a single copy of Tn*7560* was taken up and recombined into the chromosome during transformation, corresponding to the dominant amplicons for both flanks for AP_120. Sequencing of this amplicon indicated an insertion site located upstream of *fdhD*, encoding a sulfurtransferase, which matches one of the insertion sites mapped in the genome sequence of AP_1 and AP_120.

## 3. Discussion

In this study, we have identified, for the first time in *A. pleuropneumoniae*, multiple copies of a novel IS*1016*-subfamily element, designated IS*Apl4*, in the genomes of three Norwegian serovar 8 isolates obtained between 2004 and 2008 [8]. No IS*1016*-type elements (complete or partial) have previously been reported in any *A. pleuropneumoniae* genome, suggesting acquisition by horizontal transfer. There is no evidence of plasmid content—neither in the draft genomes of these isolates, nor in the complete genome of AP_123—but all three isolates carry a copy of the novel pseudo-compound transposon, Tn*7560*, comprising two copies of IS*Apl4* flanking four AMR genes, i.e., *tet*(Y), *strA*, *strB*, and *sul2.* These four AMR genes have been found on pAT205, a 159-kb plasmid isolated from the *A. towneri* strain 205, recovered from a pig in China in 2019 [34], and as part of ICE Tn*7406* carried by the *M. haemolytica* strain IMT47952, recovered from a calf in Germany in 2019 [28]. More recently, this resistance gene combination has also been identified in other Tn*7406*-related ICEs of bovine *P. multocida* and *M. haemolytica* from Germany [11,12]. Furthermore, neither sequence contains a copy of IS*Apl4*, although a different IS*1595*-type element—IS*Mha6*—was found next to a complete *sul2* gene in Tn*7406* [28].

IS*Apl4*-related (>90% at nucleotide sequence level) elements have been detected in the genomes of various *Moraxella* isolates, many having truncated *tnp* genes, likely rendering them inactive. For example, a frameshift mutation was detected in the *tnp* gene of the single IS*1016* element of *M. ovis* (GenBank accession no. CP011158) after position 37, which generates an early stop codon at positions 148–150, thereby resulting in a Tnp protein of only 49 aa. Similarly, most of the genomes of the seven *M. bovoculi* isolates from the USA collected in 2013 (GenBank accession nos. CP011376–CP011381), had single copies of the IS*1016* element with truncated *tnp* genes due to early stop codons. Only one isolate, 58069 (GenBank accession no. CP011374), contains a complete IS*1016* element with an intact *tnp* gene. These observations suggest that an IS*1016* element may have been acquired by some *Moraxella* species in the more distant past, with copies lost and/or inactivated over time. It is possible that some isolates (yet to be sequenced) still retain active copies, as in the *M. bovoculi* isolate 58069, and this may have been the source of the pseudo-compound transposon Tn*7560*, following capture of the four AMR genes from other MGEs, such as those identified in other bovine and porcine pathogens discussed above. However, where and when the formation of Tn*7560* occurred is unknown.

Acquisition of the pseudo-compound transposon Tn*7560* by the three Norwegian isolates of *A. pleuropneumoniae* is supported by the high activity of the flanking IS*Apl4* elements, with 18 or more insertions in each of the three genomes, many of which are distinct for individual isolates. Transposition bursts are common following the acquisition of a new IS, with subsequent loss, as insertions into essential genes may eliminate the affected bacterial clones. In contrast, remnants of inactive elements are commonly seen within the genomes of surviving clones [16]. The observation that there are differences in the insertion site(s) of Tn*7560* in the three Norwegian *A. pleuropneumoniae* isolates may suggest independent acquisition of Tn*7560* by different isolates, or subsequent transposition of the entire pseudo-compound transposon to different genomic locations. In this regard, it is important to know that a Tn*7560*-derived TU can be generated by recombination of the two terminal IS*Apl4* copies. This TU consists of one copy of IS*Apl4* and the four resistance genes. The IS*Apl4* in the TU can then again recombine with any complete IS*Apl4* copy in the genome of the respective isolate and, thereby, change its location within the genome. As there are numerous IS*Apl4* copies per isolate available, numerous options for new locations of Tn*7560* within the same isolate exist.

The majority of IS*Apl4* insertion sites were mapped to non-coding intergenic locations in each of the three genomes. This may explain the survival of these isolates, which were all recovered from clinical cases of pleuropneumonia in swine. Some IS*Apl4* insertions were found in genes known or predicted to contribute to virulence regulation, LPS biosynthesis, and DNA repair. This suggests that there may be functional consequences beyond resistance gene mobilisation. However, further research is required to investigate the actual impact of specific truncated or inactivated gene products on bacterial fitness and virulence. The affected genes here included those involved in the production of LPS O-Ag (*wzxE*, *wzz*) [35,36,37,38], in the regulation of biofilm formation (*cpxRA*) [25,26,27], or encoding the Flp-Tad adhesion (*tadA*) [39,40]. These IS*Apl4* insertions may affect adherence and virulence of the affected isolates but are not sufficiently deleterious to result in elimination of the respective bacteria. An insertion in the *mutY* gene (encoding the DNA repair A/G-specific adenine glycosylase) [41,42] in AP_123 likely accounts for the higher abundance of SNPs seen in this isolate relative to AP_1, AP_120 and the genomes of the serovar 8 reference strain 405 [43] and clinical isolate MIDG2331 [33]. Although only one genome rearrangement relative to the syntenic genomes of strains 405 and MIDG2331 (i.e., an inversion of sequences between two closely spaced copies of IS*Apl4*) was detected in the complete closed genome of AP_123, the abundance of insertions in the three Norwegian isolates may lead to subsequent rearrangements/deletions that may affect viability and spread of IS*Apl4* by vertical transmission.

Although attempts to transfer Tn*7560* via conjugation were unsuccessful, horizontal transfer of Tn*7560* to a new strain of *A. pleuropneumoniae* was achieved by natural transformation, indicating that this element may further spread the associated AMR genes to other isolates of this bacterial species. However, Tn*7560* has—to the best of our knowledge—not yet been detected in other clinical isolates of *A. pleuropneumoniae*. The Tn*7560*-associated AMR genes conferred resistance to tetracycline (MIC ≥ 8 µg/mL), streptomycin (MIC ≥ 256 µg/mL) and sulfisoxazole (MIC ≥ 512 µg/mL) to all isolates in which it was present (Table 2). Based on the CLSI-approved clinical breakpoints, *A. pleuropneumoniae* isolates with tetracycline MICs of ≥2 µg/mL are considered tetracycline-resistant. Although no *A. pleuropneumoniae*-specific CLSI-approved clinical breakpoints exist, at least 16-fold and at least 4-fold increases in the MICs of sulfisoxazole and streptomycin of the transformants in comparison to the “empty” recipient strongly suggest the activity of the respective resistance genes. Moreover, sulfisoxazole and streptomycin concentrations of 512 and 256 µg/mL cannot be reached in the lung of swine by regular dosing, suggesting isolates with such high MICs should be considered as resistant. As the *tet*(Y) gene has rarely been found in *Pasteurellaceae*, any tetracycline-resistant *A. pleuropneumoniae* isolates should be tested by PCR using the *tet*(Y)-specific primers designed in this study to facilitate further epidemiological studies on the spread of this *tet* gene. Furthermore, molecular surveillance for IS*Apl4* is also warranted, as this novel element may facilitate acquisition and spread of other AMR genes, as has been documented for the previously characterised IS*Apl1* element, which, since its first detection in isolates of *A. pleuropneumoniae* [21], has been associated with the spread of the colistin resistance gene *mcr*-1 [44].

## 4. Materials and Methods

### 4.1. Bacterial Isolates and Antimicrobial Susceptibility Testing

*A. pleuropneumoniae* isolates were cultured at 37 °C with 5% CO_2_ on Columbia agar (BD, Franklin Lakes, NJ, USA) supplemented with 0.01% nicotinamide adenine dinucleotide (NAD). The serovar 8 *A. pleuropneumoniae* isolates were cultured from pneumonic lungs of pigs in Norway, with AP_1 isolated in 2004, and AP_120 and AP_123 isolated in 2008, but each from a different part of Norway [8]. These isolates were sent to Imperial College London for further analysis. Isolate AP_123 was also sent to the Statens Serum Institut, Denmark, to generate a complete closed genome sequence. The UK serovar 8 strain MIDG2331 [33] was used to test the transfer of the pseudo-compound transposon Tn*7560* by natural transformation, and the NAD-independent MIDG2331∆*ureC*::*nadV* mutant [45] was used to test the transfer of Tn*7560* from AP_1 by conjugation. The susceptibility of isolates and transformants to tetracycline, streptomycin, and sulfisoxazole was analysed by the broth microdilution method according to CLSI recommendations [31]. The reference strain *A. pleuropneumoniae* ATCC 27090 served as the quality control strain. *A. pleuropneumoniae*-specific CLSI-approved clinical breakpoints were used, as far as available, to classify the isolates as resistant, intermediate or susceptible based on the MICs obtained.

### 4.2. Genome Analysis and Identification of ISApl4 Insertions

Draft whole-genome sequences for AP_1, AP_120, and AP_123 (ENA accession nos. ERR6510189, ERR6510049, and ERR6510052, respectively) were generated as part of a previous study [8]. ResFinder v4.7.2 [46] was used to identify contigs containing the AMR genes in each genome. As all AMR genes, i.e., *tet*(Y), *sul2*, *strA* and *strB*, were on a single contig of the same size in each genome, these three contigs were aligned using the ClustalW feature of MacVector v15.5.4 (MacVector Inc, Cambridge, UK) and analysed by a blastn search against the NCBI nr/nt database to identify sequences with the highest identity (https://blast.ncbi.nlm.nih.gov/Blast.cgi (accessed on 29 September 2025)). As the majority of the contig sequence matched various possible plasmids, but no plasmid replication genes were present, the ends of the contigs were examined to determine if overlaps to other contigs could be found. The presence of a 14-bp IR at either end of the contig was identified, along with sequences matching either the 5′ or 3′ end of an IS*1016*-type IS, respectively. The 14-bp IR sequence was used to search each of the three genomes for other contigs where it was present.

For each contig in which the IR sequence was located, near either the 5′ or 3′ (or in some cases both) termini, the proximal sequences were analysed to map the genomic location in comparison with the closed genomes of serovar 8 strains 405 (GenBank accession no. CP078508) and MIDG2331 (GenBank accession no. LN908249), and to identify paired contigs containing the 5′ and 3′ ends of the IS element. For each paired contig, the sequences immediately proximal to the IRs were analysed to identify possible DR sequences generated by the insertion. Using the genomic sequence data, we also applied ISMapper [47] with the IS*ApI4* as query to identify sites and orientations of IS insertions in *A. pleuropneumoniae,* using default settings.

### 4.3. Generation of a Closed Genome of AP_123

To obtain a closed high-quality genome of AP_123, DNA from a full 1 µL loop of colonies was extracted with the GenFind v3 kit (Beckman Coulter, Amersham, UK) using a DynaMag-2 magnet (Thermo Fisher Scientific, Loughborough, UK). A library was prepared using the Rapid Barcoding Sequencing Kit (SQK-RBK004) and sequenced in a R10.3 flow cell (FLO-MIN111) with a MinION Mk1B (Oxford Nanopore Technologies, Oxford, UK). Using Guppy v4.2.2 (Oxford Nanopore Technologies, Oxford, UK), raw fast5 reads were base-called to fastq format in ‘high-accuracy’ configuration, demultiplexed and quality filtered to a minimum q8 using NanoFilt [48]. Illumina data were trimmed with Trimmomatic v0.36 [49] to remove low-quality (Q ≤ 20) read ends, and then a hybrid *de novo* genome assembly was obtained with Unicycler v0.4.8-beta [50] using default parameters.

### 4.4. SNP Analysis of the ISApl4-Containing Isolates

To investigate the relatedness of the four IS*Apl4*-containing isolates (AP_1, AP_120, AP_123, 405), SNPs were identified in the core genome using the raw sequencing data aligned to the *A. pleuropneumoniae* MIDG2331 reference chromosome (GenBank accession no. LN908249) [33]. Only high-quality SNPs were retained by exclusion of any site if a minimum coverage of 10 was not met or if the variant was present in less than 90% of the base calls of individual isolates.

### 4.5. Analysis of ISApl4 Activity

RT-PCR was used to determine the activity of the *tnp* gene in the *A. pleuropneumoniae* isolates. Briefly, cultures were grown in Columbia-NAD broth with/without 5 µg/mL tetracycline to OD_600_ = 0.4, and RNA was extracted using the RNeasy Mini Kit (Qiagen Ltd., Manchester, UK), as per the manufacturer’s instructions. Samples were treated with the TURBO DNA-free Kit (Thermo Fisher Scientific) to remove residual genomic DNA. Approximately 600 ng RNA per sample were used as template for amplification using the OneStep RT-PCR kit (Qiagen, Hilden, Germany), according to the manufacturer’s instructions, with primers ISApl4_tnp_for and ISApl4_tnp_rev (Table 3). As negative controls, the RNA samples were also subjected to PCR without RT to verify the removal of all gDNA. The resulting 429-bp PCR products were visualized with ethidium bromide after separation by agarose gel electrophoresis.

Inverse PCR was used to test for the generation of circular intermediates during transposition, using gDNA extracted from an aliquot of the cultures prepared above (i.e., culture for RT-PCR). Outward-facing primers ISApl4_inv1 and ISApl4_inv2 would generate a product of 718 bp if the IS*Apl4* sequence formed a circular intermediate joined by the overlap of the 8-bp DR sequences generated at each insertion site.

To test for possible transfer of Tn*7560* to another *A. pleuropneumoniae* isolate via conjugation, we used the MIDG2331∆*ureC*::*nadV* mutant as a recipient strain in a mating experiment, as the presence of ICE*Apl1* in MIDG2331 [51] supplies the conjugal transfer machinery that can mediate two-way exchange (retro-transposition) of DNA between isolates, and the presence of the *nadV* gene allows counterselection for transconjugants containing the pseudo-compound transposon on media without supplemental NAD. Conditions for the mating experiment were as previously described [51,52], with selection for NAD-independence and streptomycin-resistance using Columbia agar containing 200 µg/mL streptomycin.

To test for horizontal transfer of Tn*7560* to another *A. pleuropneumoniae* isolate by natural transformation, we used MIDG2331 as a recipient, which is competent for natural transformation [53], as the recipient for uptake of gDNA extracted from each of the three Norwegian isolates (AP_1, AP_120, and AP_123), using the plate transformation assay previously described [53], with selection for transformants on Columbia-NAD agar containing 200 µg/mL streptomycin. The resulting transformants were tested by PCR for the presence of the IS*Apl4 tnp*, *tet*(Y), *sul2* and *strA*-*strB* genes (see Table 3 for primer sequences) to confirm the presence of the pseudo-compound transposon. Selected clones were tested for MICs of tetracycline, streptomycin and sulfisoxazole by broth microdilution (Table 2). Linker PCR was performed, as previously described [54]. Oligos 254 and 256 were used to generate the linker to be ligated to blunt-end restriction fragments generated by DpnI digestion of the genomic DNA. Transposon-flanking DNA fragments were then amplified using an outward-facing transposon-specific primer (tetY_5′_out or sul2_up_out) and a linker-specific primer, oligo 258. The transposon insertion sites were then determined by sequencing of the purified linker-PCR amplicons using the outward-facing ISApl4_inv1 (for tetY_5′_out amplicons) or ISApl4_inv2 (for sul2_up_out amplicons).

## 5. Conclusions

In conclusion, we have identified a novel IS*1016* element, IS*Apl4*, and the associated MDR-encoding pseudo-compound transposon, Tn*7560*, which was shown to be active in isolates of *A. pleuropneumoniae*. Results indicate acquisition and transposition bursts leading to large numbers of IS*Apl4* copies. Vertical transmission of this element may be mitigated by the introduction of deleterious mutations following further intrachromosomal spread of this IS, and/or genomic rearrangements/deletions, leading to elimination of affected clones. However, horizontal spread of IS*Apl4* to other isolates of *A. pleuropneumoniae* is possible by natural transformation. Furthermore, acquisition into other MGEs may result in the spread to other species found within the same host environment.

## Figures and Tables

**Table 1:** Characteristics of 35 of the 39 IS*Apl4* integration sites in the genome of AP-123.

IS*Apl4* Copy No.	8-bp DR	Genomic Context
1	AAAAAAAA	non-coding region
2	AAAAAGAA	non-coding region
3	AAACAAAA	intragenic (DMT family transporter/EamA domain-containing protein)
4	AAACATAA	non-coding region
5	AAATTTAA	non-coding region
6	AAATTTTT	non-coding region
7	AAATTTTT	non-coding region
8	AACAATTA	intragenic (*tadA*; CpaF family protein/tight adherence protein A)
9	AACTAAAA	non-coding region
10	AAGTTTTA	non-coding region
11	AATAAATA	intragenic (sulfatase N-terminal domain-containing protein)
12	AATTAATT	intragenic (tetratricopeptide repeat protein)
13	ATAAAAAA	intragenic (hypothetical protein)
14	ATAATTTA	non-coding region
15	ATGATAAA	non-coding region
16	ATGATTTT	intragenic (*udk*; uridine kinase)
17	ATTAAATA	non-coding region
18	CAATTTAA	intragenic (hypothetical protein)
19	GATATTTT	intragenic (*phoB*; phosphate regulon transcriptional regulator)
20	TAAATAAA	intragenic (Tex family protein/predicted transcriptional accessory protein)
21	TAAATGAA	non-coding region
22	TAAATTAA	intragenic (*manX*; PTS mannose transporter subunit IIAB)
23	TAATAAAA	intragenic (LPS O-antigen chain length determinant protein, WzzB/FepE family)
24	TAATTTAA	intragenic (N-6 DNA methylase/class I SAM-dependent DNA methyltransferase)
25	TAATTTTT	intragenic (*wzxE*; lipid III flippase WzxE/lipopolysaccharide biosynthesis protein)
26	TATCTTAA	non-coding region
27	TTAAAAAT	non-coding region
28	TTAATAAA	non-coding region
29	TTAATAAA	non-coding region
30	TTAATTTA	non-coding region
31	TTAGTTTT	intragenic (*mutY*; A/G-specific adenine glycosylase)
32	TTATTAAA	intragenic (DUF1523 domain-containing protein)
33	TTTAATTA	intragenic (hypothetical protein)
34	TTTTAAGG	non-coding region
35	TTTTATAA	non-coding region

DR, direct repeat; inverted repeat left (IRL): GGGGCTGTACTAGA; single mismatch for copy no. 23 GGGG**A**TGTACTAGA; inverted repeat right (IRR): GGGGCTGTACTAGA; All sequences, except IRR, are shown in the 5′ → 3′ orientation. The IRR sequences are presented in the reverse complementary orientation to better illustrate the identity with the IRL sequences. Sequences of left and right flanking regions can be found in Table S1.

**Table 2:** MICs of relevant antimicrobial agents for *A. pleuropneumoniae* isolates containing Tn*7560*.

Isolate	Tetracycline MIC	Sulfisoxazole MIC	Streptomycin MIC
AP_1	16	>512	>256
MIDG2331/AP_1	16	>512	>256
AP_120	16	>512	>256
MIDG2331/AP_120	16	>512	>256
AP_123	8	>512	>256
MIDG2331/AP_123	8	>512	>256
MIDG2331	2	32	64

MIC; minimal inhibitory concentration in µg/mL. Strain MIDG2331 carries *tet*(B) as part of the ICE*Apl1* insertion [33], but no other AMR genes.

**Table 3:** Sequences of primers used in this study.

Primer Name	Sequence 5′ to 3′	Target/Purpose
ISApl4_tnp_for	TTGAAGTTACTGCTCGTTCGGC	IS*Apl4 tnp*; 429-bp amplicon
ISApl4_tnp_rev	CGTTGATGTGATTATGGTCTTTTGC	
ISApl4_inv1	TGAGAATTTCTGGTCGCAAGCC	IS*Apl4* circular form (718-bp amplicon) and transposon insertion site mapping
ISApl4_inv2	GCCGTTGATGTGATTATGGTCTTTTG	
tetY_for	TATGGACGGCGGATTATTTTGCTG	*tet*(Y) gene; 530-bp amplicon
tetY_rev	GTGCCAATATCCCAATTCAAGCG	
sul2_for	TCAACATAACCTCGGACAGTTTCTC	*sul2* gene; 212-bp amplicon
sul2_rev	GGGAATGCCATCTGCCTTGAGC	
strA_for	GATTTTGTTTTTCGACGTGGTGAC	*strA*-*strB*; 796-bp amplicon
strB_rev	GTGTCCGCAATGAGAACAGG	
oligo 254	CGACTGGACCTGGA	Generation of linkers
oligo 256	GATAAGCAGGGATCGGAACCTCCAGGTCCAGTCG	
oligo 258	GATAAGCAGGGATCGGAACC	Linker-specific primer for linker PCR
tetY_5′_out	CATCAAGCGCGACAACAATGAG	Transposon-specific primer for linker PCR
sul2_up_out	TCTGGAAGCAAAGCAAGGAAAGC	

## Data Availability

All data presented in this study are available in the text, figures and tables of the main article. Whole-genome sequences of the investigated *A. pleuropneumoniae* isolates are available at GenBank within BioProject PRJEB47034 (https://www.ncbi.nlm.nih.gov/genbank/ (accessed on 29 September 2025)).

## Supplementary Materials

The following supporting information can be downloaded at: https://www.mdpi.com/article/10.3390/antibiotics14101021/s1, Table S1. Detailed characteristics of 35 of the 39 IS*Apl4* integration sites in the genome of AP-123.

## Abbreviations

The following abbreviations are used in this manuscript:

AMR	Antimicrobial resistance
CLSI	Clinical and Laboratory Standards Institute
DR	Direct repeat
gDNA	Genomic DNA
ICE	Integrative and conjugative element
IR	Inverted repeat
IS	Insertion sequence
LPS	Lipopolysaccharide
MGE	Mobile genetic element
MDR	Multidrug resistance
MIC	Minimal inhibitory concentration
NAD	Nicotinamide adenine dinucleotide
PCR	Polymerase chain reaction
RT-PCR	Reverse transcription polymerase chain reaction
SNP	Single nucleotide polymorphism
TU	Translocatable unit
UK	United Kingdom
USA	United States of America
