# Supplementary material for: ISApl4, a New IS1595 Family Insertion Sequence Forming a Novel Pseudo-Compound Transposon That Confers Antimicrobial Multidrug Resistance in Actinobacillus pleuropneumoniae
Source: Antibiotics (Basel). 2025 Oct 14;14(10):1021. doi: 10.3390/antibiotics14101021 (PMC12561309; doi:10.3390/antibiotics14101021)
Supplement: Supplementary file 1 [file antibiotics-14-01021-s001.zip › antibiotics-3881028-supplementary.pdf]

Table S1. Detailed characteristics of 35 of the 39 IS*Apl4* integration sites in the genome of AP-123.

IS*Apl4* copy no.	Left flanking region	IRL	8-bp DR	IRR	Right flanking region
1	GTTTTCCAGTTAATA	GGGGCTGTACTAGA	AAATTTTT	GGGGCTGTACTAGA	AGTTTGAATTCGGTT
2	ACTTTTGTTATAATA	GGGGCTGTACTAGA	AAACAAAA	GGGGCTGTACTAGA	TGTAGCATTTGAGTA
3	CCCATAACTGCAATT	GGGGCTGTACTAGA	CAATTTAA	GGGGCTGTACTAGA	TTATTTACTATTATC
4	CAAAAATGCTTTACA	GGGGCTGTACTAGA	TAAATTAA	GGGGCTGTACTAGA	TTTATTATTTACAAT
5	CACCTCTGGTCTGAA	GGGGCTGTACTAGA	TAATTTTT	GGGGCTGTACTAGA	ACATTGTACCTGATT
6	ATAGGTTTCCTCAAA	GGGGCTGTACTAGA	AACAATTA	GGGGCTGTACTAGA	TTCTTGCTCAAGAAT
7	ATTTTGTAATGAATA	GGGGCTGTACTAGA	AAATTTTT	GGGGCTGTACTAGA	AAAGTAATTTGGAGT
8	ATTATCCGGACCATT	GGGGCTGTACTAGA	AACTAAAA	GGGGCTGTACTAGA	TATCGTTTACACCCT
9	AAGCCCTATAAAGGA	GGGGCTGTACTAGA	TTTAATTA	GGGGCTGTACTAGA	TGTTATTGCTCGATC
10	TGTTTAATGTAAATA	GGGGCTGTACTAGA	TATCTTAA	GGGGCTGTACTAGA	AGTTTATTTGTATAA
11	AACAGTTCACGTTGT	GGGGCTGTACTAGA	GATATTTT	GGGGCTGTACTAGA	TGCGCAATTTTCGCA
12	TAAATTCATTTGGTA	GGGGCTGTACTAGA	ATAATTTA	GGGGCTGTACTAGA	AATAAAGTACTTGGT
13	ACACCAGTATCTTTA	GGGGCTGTACTAGA	TTAAAAAT	GGGGCTGTACTAGA	AATTTGAATACTACT
14	TTTTTACTAAATATA	GGGGCTGTACTAGA	AAAAAAAA	GGGGCTGTACTAGA	TTTTTTTAAAATCAT
15	TACTAAGGTGCGTTT	GGGGCTGTACTAGA	TTAGTTTT	GGGGCTGTACTAGA	TGAATCGCTATTCCG
16	ATACCTTCGAGAATA	GGGGCTGTACTAGA	ATGATTTT	GGGGCTGTACTAGA	TTTAGGTGTGAAATT
17	TCTTTTCTAGGAATA	GGGGCTGTACTAGA	TAAATGAA	GGGGCTGTACTAGA	TCAAGCAAAACGAAT
18	ATTCACATCGGTAAA	GGGGCTGTACTAGA	TTTTAAGG	GGGGCTGTACTAGA	TATCAATATCTTTTT
19	GCGTTACCTTCCAGA	GGGGCTGTACTAGA	AAAAAGAA	GGGGCTGTACTAGA	ATTAGCTTAAGCAGC
20	CATTATGCTGCTTAA	GGGGCTGTACTAGA	TTAATTTA	GGGGCTGTACTAGA	TACAGCGAAACTTCC
21	TGCTAATAAATATTA	GGGGCTGTACTAGA	TTATTAAA	GGGGCTGTACTAGA	TAATGGTATACATCT
22	TTAAGCTGTTTTTCT	GGGGCTGTACTAGA	TTAATAAA	GGGGCTGTACTAGA	TCCATCATATACTGA
23	TTTACGTAAAGAATA	GGGG**<u>A</u>**TGTACTAGA	TAATTTAA	GGGGCTGTACTAGA	TTATTGGTTCAGAGT
24	AAAACGATTCTATTA	GGGGCTGTACTAGA	ATGATAAA	GGGGCTGTACTAGA	AATCTACTTAGCAAT
25	CCATACCGCTGAACT	GGGGCTGTACTAGA	TAAATAAA	GGGGCTGTACTAGA	TTTCGCGCATATTGC
26	TCTAAAATACAAAAA	GGGGCTGTACTAGA	TAATAAAA	GGGGCTGTACTAGA	TAAGGAATAAATGAA
27	ACTCCGTATTGTCAA	GGGGCTGTACTAGA	ATAAAAAA	GGGGCTGTACTAGA	ATATAAAAAGAAAAT
28	AGAATAGGCATTATT	GGGGCTGTACTAGA	TTAATAAA	GGGGCTGTACTAGA	TATAATTAGGTATGA
29	TTTATAAAGGGCTTT	GGGGCTGTACTAGA	TTTTATAA	GGGGCTGTACTAGA	TATAAAAGGTATAAT
30	ATTAAATCAGCATAA	GGGGCTGTACTAGA	AATTAATT	GGGGCTGTACTAGA	ATTTTATCTGTGGAT
31	ACCAAACCCCATATA	GGGGCTGTACTAGA	AAGTTTTA	GGGGCTGTACTAGA	TTTTGTTGCCAAGGT
32	AAACAACTGGTGCAA	GGGGCTGTACTAGA	ATTAAATA	GGGGCTGTACTAGA	TTACGGCTGGCGAAT
33	TTAAAAGCCCCTCAA	GGGGCTGTACTAGA	AATAAATA	GGGGCTGTACTAGA	TTTAAACTTAATTAT
34	TTTGGACAATGTTTA	GGGGCTGTACTAGA	AAATTTAA	GGGGCTGTACTAGA	ACATTGTAACTGCCT
35	TTATTAAATTTGATA	GGGGCTGTACTAGA	AAACATAA	GGGGCTGTACTAGA	AGCGTTTTATCAAGG

IRL, inverted repeat left; IRR, inverted repeat right; DR, direct repeat; All sequences, except IRR, are shown in the 5′→ 3′ orientation. The IRR sequences are presented in the reverse complementary orientation to better illustrate the identity with the IRL sequences. The single mismatch in one of the IRL sequences is shown in bold and underlined.
